# Molecular detection and genetic characterization of human metapneumovirus strains circulating in Islamabad, Pakistan

**DOI:** 10.1038/s41598-022-06537-5

**Published:** 2022-02-18

**Authors:** Yasir Arshad, Muhammad Suleman Rana, Aamer Ikram, Muhammad Salman, Uzma Bashir Aamir, Syed Sohail Zahoor Zaidi, Muhammad Masroor Alam, Salmaan Sharif, Shahzad Shaukat, Adnan Khurshid, Rabia Hakim, Ghulam Mujtaba, Massab Umair, Sadia Sattar, Nazish Bostan

**Affiliations:** 1 National Institute of Health, Islamabad, Pakistan; 2 COMSATS University Islamabad, Islamabad, Pakistan

**Keywords:** Virology, Microbiology, Molecular biology

## Abstract

Lower respiratory illness is one of the leading causes of death among children in low- and high-income countries. Human metapneumovirus (hMPV) is a key contributor to respiratory illnesses commonly reported among children and causes serious clinical complications ranging from mild respiratory infections to severe lower respiratory tract anomalies mainly in the form of bronchiolitis and pneumonia. However, due to the lack of a national surveillance system, the clinical significance of hMPV remains obscure in the Pakistani population. This study was conducted to screen throat swabs samples collected from 127 children reported with respiratory symptoms at a tertiary care hospital in Islamabad. Out of 127, 21 (16.5%) samples were positive for hMPV with its genotype distribution as A2a (10%), A2b (20%), B1 (10%), and B2 (60%). Phylogenetic analysis showed that the hMPV viruses were closely related to those reported from neighboring countries including India and China. This work will contribute to a better understanding of this virus, its diagnosis, and the handling of patients in clinical setups. Further studies at a large-scale are warranted for a better understanding of the disease burden and epidemiology of hMPV in Pakistan.

## Introduction

Human metapneumovirus (hMPV) was first discovered as a novel pathogen among children with acute lower respiratory illness in the Netherlands in 2001^1,2^. Based on morphological studies of the virion and genome organization, hMPV has been classified as *Metapneumovirus* genus of family *Pneumoviridae*^1,3,4^, measuring 150–600 nm in size, pleomorphic with envelope spikes as projections ranging 13–17 nm^1,2^. It has a negative-sense single-stranded RNA genome, reported as one of the leading causes of lower respiratory tract illnesses among children worldwide, and is associated with infections among immunocompromised hosts and other underlying morbidities^5^. It also causes a broad range of infections among children from upper respiratory air tract infections to severe lower respiratory tract illness leading to pneumonia and bronchiolitis^6^. In the respiratory tissues, hMPV causes inflammation and necrosis of the epithelial lining of bronchioles and result in sloughing^7^.

Various reports have shown positivity of the hMPV from 8.5 to 11% in Canada and Argentina respectively^8^. On the other hand, the relatively low prevalence was reported in previous studies from Pakistan i.e., 5.2%^9^ and 7% respectively^10^.

**Figure 1:**
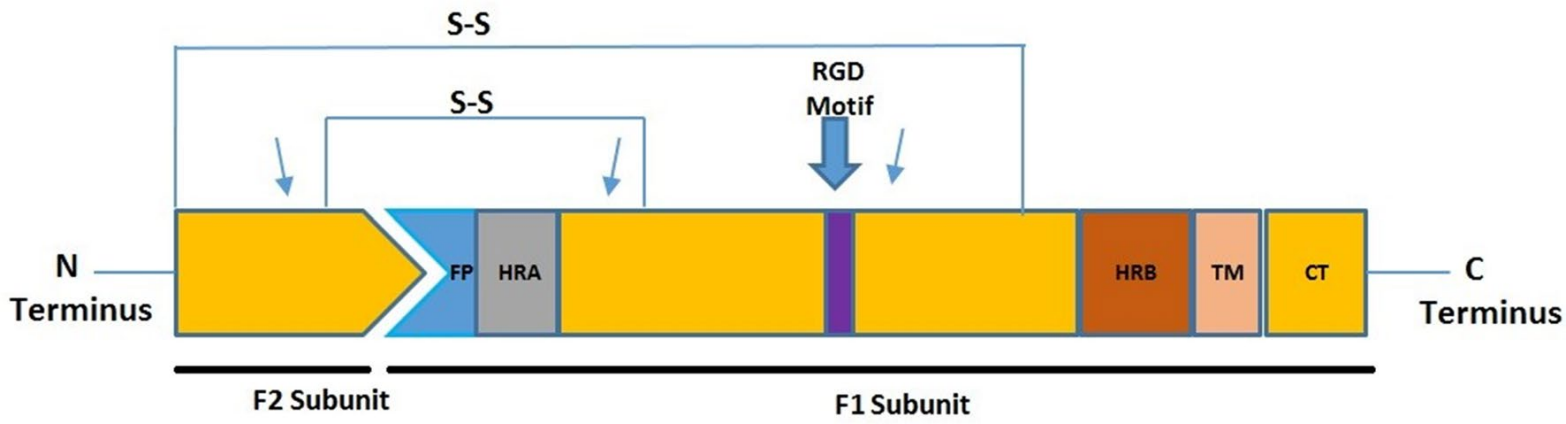
Schematic representation of the cleaved hMPV fusion protein. *FP* fusion peptide, *HRA* heptad repeat A, *HRB* heptad repeat B, *TM* transmembrane domain, *CT* cytoplasmic tail. The approximate location of a conserved RGD motif (residues 329–331) is indicated as a magenta box. Arrows indicate the three *N*-linked glycosylation sites. The location of two disulfide bonds that connect the F1 and F2 protein subunits are shown as S–S.

hMPV F protein plays an important role in antigenicity during virus entry which facilitates broad cross-lineage neutralization and protection. Members of the family *Pneumoviridae* have less identical but structurally similar fusion proteins which are produced as the inactive precursors F_0_. These inactive precursors are cleaved by host cell proteases and form two subunits; F1 forming C-terminus and F2 which forms N-terminus subunit. This activated fusion protein in combination with the attachment protein binds the viral membrane to the cell membrane of the host. F gene codes for 539 amino acids protein which is 81% identical to the Fusion protein of APV (Avian pneumovirus) and is glycosylated having three potential N glycosylation sites. 12 of fourteen cysteine residues are in the F1 subunit whereas two are located in the F2 subunit and 8 residues are conserved amongst entire paramyxoviruses. hMPV constitutes one cleavage site, unlike RSV which comprises two cleavage sites. There is a primary sequence in hMPV F protein that contains 3 hydrophobic domains; a signal peptide which is located on the N-terminal of the subunit F2 and fusion anchor domain at N terminal and membrane anchor domain at C terminal in the F1 subunit^3^. F protein constitutes two Heptad Repeat Domains; HRA and HRB which are necessary for the fusion of the virus to the target membrane and located in the subunit F1 and fusion protein^11^ (Fig. 1). Therefore, by using the F protein gene we demonstrated the clinical and molecular diversity, epidemiological features, and prevalence of hMPV circulating in Islamabad, Pakistan during 2015.

## Materials and methods

### Ethics statement

The study was approved by the internal review board of the National Institute of Health (NIH) Islamabad. Informed consent was obtained using a designated proforma, from all parents/guardians of children enrolled in this study (Consent proforma attached). All experiments were performed by approved guidelines and regulations by the Centers for Disease Control and Prevention, USA.

### Sample collection

The case definition of severe acute respiratory infection (SARI) standardized by the World Health Organization (WHO) was used to enroll patients in this study. According to this definition, patients reported with sudden onset of fever (> 38 °C), cough, sore throat, and shortness of breath that required hospital admission within 7 days were termed as SARI^12^.

The samples were collected from January–December 2015 from the Pakistan Institute of Medical Sciences Hospital, Islamabad (PIMS). The clinical data were recorded on a standardized ILI/ARI/SARI patient history form (Patient History form attached) recommended by CDC and WHO. The patient’s age was ≤ 5 years exhibiting symptoms of severe acute respiratory illness.

### Molecular characterization and phylogenetic analysis of hMPV

Nucleic acid extraction was performed using QIAamp viral RNA mini kit (QIAGEN, Hilden, Germany) and RNA was eluted in 60 µl of Tris–EDTA buffer. Samples were initially screened for the presence of hMPV RNA by real-time PCR using primers and probes based on F-gene (Supplementary Table 1).

For genotype identification, cDNA was synthesized by two-step PCR that amplified partial F-gene segment of 750 bp using primers (Supplementary Table 1)^13^ and annealing temperature of primers used were 45 °C and 48 °C whereas amplification was confirmed by gel electrophoresis (Supplementary Appendix 1). Samples positive on gel electrophoresis were sequenced by respective forward and reverse primers using Sanger sequencing and run onto ABI Prism 3130xl Genetic Analyzer (Applied Biosystems, Foster City, CA, USA). Cleaning and editing of raw sequence data was performed using Sequencher v4.9 (Gene Codes, Ann Arbor, MI, USA). Multiple sequence alignment was performed using CLUSTAL W program and maximum likelihood tree was reconstructed using MEGA v5.0.^14^. Maximum likelihood tree was obtained by applying the Kimura-2 Parameter model for nucleotide substitution and evolutionary distances were calculated using the p-distance method.

### Statistical analysis

Different variables related to suspected patients were analyzed using the SPSS package (version 20.0, IBM) for analyzing the p-value. We analyzed the distribution of hMPV positive patients for gender and different age groups. The clinical signs of all positive and negative patients were also statistically compared.

## Results

A total number of 127 samples were tested using real-time PCR for the detection of hMPV. Twenty-one (16.5%) were found positive during the study period from January to December 2015. The mean age of patients included in this study was < 5 years. Among 21 hMPV positive samples, 12 were male and 9 were female children. hMPV positive percentage was the highest among children above 2 years of age, whereas the lowest among younger children less than 2 years of age. hMPV infection was found equally distributed among male and female patients.

The clinical symptoms frequently associated with hMPV positive cases were cough, sputum, wheezing, and vomiting followed by fever, shortness of breath, and tachypnea (Table 1). Examination of X-ray radiography reports available for 6 hMPV positive children showed infiltration of lungs in two children and consolidated lungs found in one child, all three were admitted to Intensive Care Unit. The remaining three hMPV positive children had normal chest pictures. Clinical symptoms of respiratory illness were also present in hMPV negative children.

**Table 1:** Clinical symptoms among hMPV tested patients.

Chi square analysis			
Total no of cases (127)	hMPV positive (21)	hMPV negative (106)	P-values
	No. (%)	No. (%)	
**Gender**			
Male n (68)	12 (17.6)	56 (82.3)	0.1
Female n (59)	9 (15.2)	50 (84.7)	
**Age groups**			
< 1 year (28)	4 (14.2)	24 (85.8)	3.2
1–2 year (34)	3 (8.8)	31 (91.2)	
> 2–3 (17)	4 (23.5)	13 (76.5)	
> 3–4 year (18)	3 (16.7)	15 (83.3)	
> 4–5 year (30)	7 (23.3)	23 (76.7)	
**Clinical signs**			
High fever > 38			
Yes (120)	21 (17.5)	99 (82.5)	2.6
Cough			
Yes (21)	6 (28.5)	15 (71.4)	0
Nasal congestion			
Yes (104)	18 (17.3)	86 (82.7)	0.2
Sore throat			
Yes (106)	19 (18)	87 (82)	1.0
Wheeze			
Yes (100)	19 (19)	81 (81)	2.4
Tachypnea			
Yes (96)	18 (18.8)	78 (81.2)	1.5
Short breath			
Yes (111)	20 (18.0)	91 (81.9)	1.7
Vomiting			
Yes (5)	1 (20)	4 (80)	0.04^(S)^*
Sputum			
Yes (5)	1 (20)	4 (80)	1.3

*Significant value.

**Figure 2:**
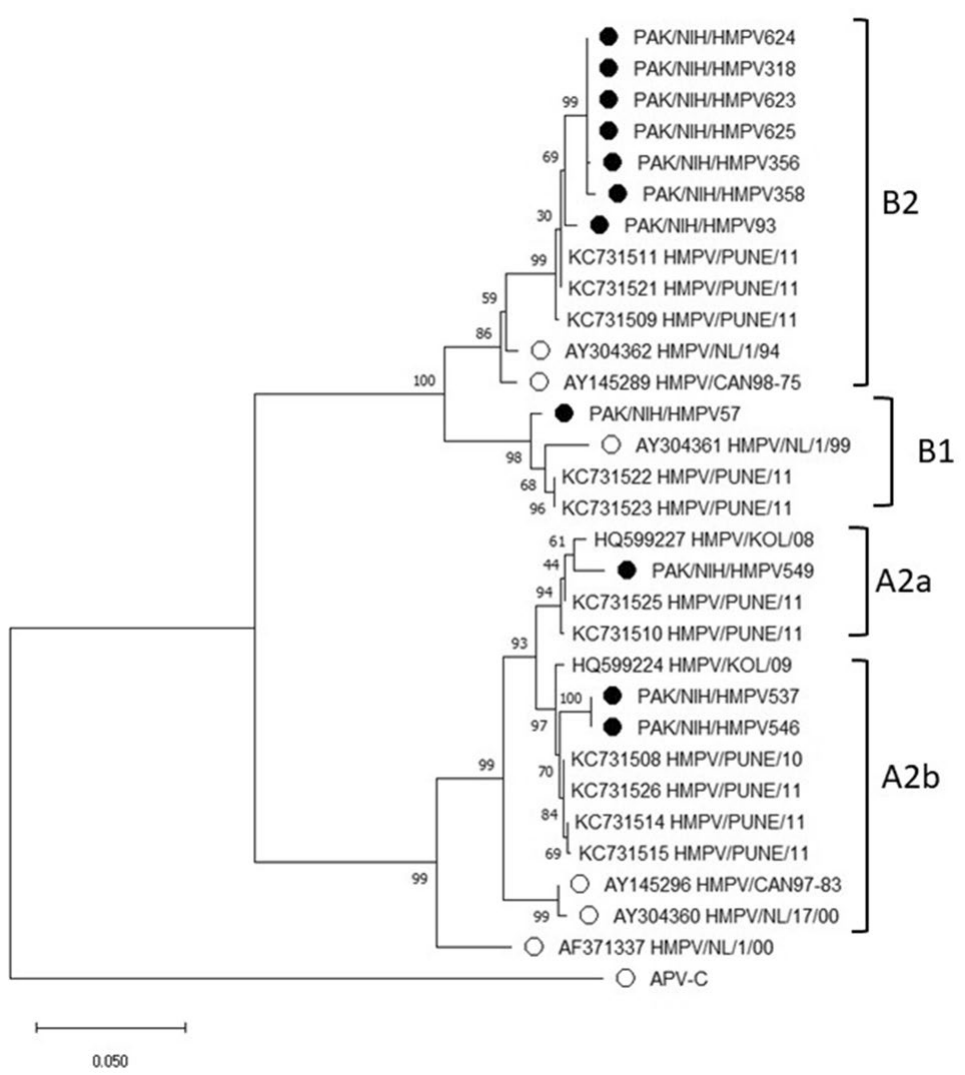
Phylogenetic analysis of partial F gene of 11 hMPV strains identified in this study (filled circles) Empty circles represents the prototypes for the groups or sub-groups using the Maximum Likelihood method.

hMPV genotypes were confirmed using nucleotide sequencing and BLAST analysis. Both genogroups A and B were found with genogroup B being the most frequent (70%) followed by genogroup A (30%). The hMPV positive strains detected in this study clustered with representative viruses from groups A2, B1, and B2 whereas no strain of A1 was found. The sequences derived from the study were deposited to GenBank under accession numbers MK144791–MK144800. Among the hMPV positive strains A2a (10%), A2b (20%), B1 (10%), and B2 (60%) subgroups were confirmed on phylogenetic analysis with respective prototype strains (Fig. 2). Sub-group A2 was found co-circulating with B1 and pre-dominant sub-group B2. Nucleotide sequence analysis showed homology between A and B strains ranging 83.6% whereas nucleotide homology among B strains was 93.1% (Table 2). Sub-group B2 was the most frequently detected group of hMPV among representative children in this study which reflect its predominant circulation in Islamabad.

**Table 2:** Percentage nucleotide and amino acid identity for F protein in the Pakistan hMPV genotypes.

Gene/protein	Genotype/sub-lineage	Nucleotide identity (%)	Amino acid identity (%)
F	Overall	83.6	95.4
	Within A2	98	100
	Within A2b	100	100
	Within B	93.1	99.4
	Within B2	99.5	99.6

In this study, it was found that 9.5% of samples were detected hMPV positive during February, 23.8% detected in November whereas 52.3% samples were detected in December. All sub-groups were present in hMPV positive samples in December except B1 whereas only B1 and B2 were found during January and November. Detection of hMPV in the first and last quarters of the year reflects its transmission in the winter season.

### Protein analysis

**Figure 3:**
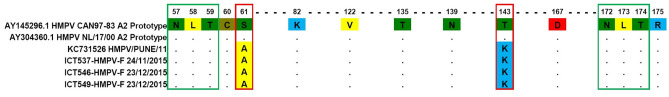
Sequence alignment of hMPV A2 genotype. The two *N*-glycosylation sites are highlighted with green squares and substitutions are highlighted with red squares.

Amino acid alignments of the hMPV F gene were compared to those of the prototype isolates from the Netherlands and Canada (Fig. 3). Cysteine residues were conserved in all Pakistani hMPV strains at positions 60 and 182. Group-specific amino acid residue at positions 122(V/I), 135(T/N), 139(N/G), 167(D/E), 175(R/S), 179(K/R), 223(T/N), and 233(N/Y) differentiated between genogroups A and B. Further amino acid substitutions at various positions were exclusive to subgroups A1 (amino acids [aa] 61, 82, 143), A2 [aa 61(A), 143(K), 185(D)], and subgroups B1 [aa 46(N), 143(Q), 179(R)] and B2 [aa 143(T)].

**Figure 4:**
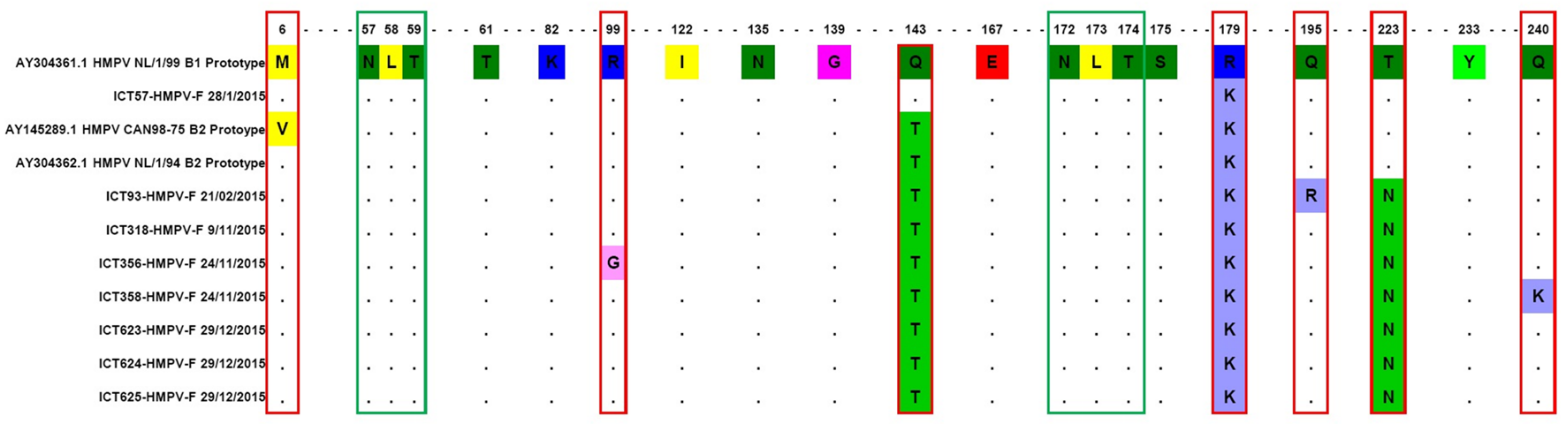
Sequence alignment of hMPV B2 genotype. The two *N*-glycosylation sites are highlighted with green squares and substitutions are highlighted with red squares.

In hMPV A genotypes, unlike the A2 prototype strains from Netherlands and Canada, all Pakistani hMPV viruses had alanine instead of serine at amino acid position 61 and lysine instead of threonine at position 143. Among HMPV B genotypes samples, ICT 356/15 had Arginine to Glycine substitution at amino acid position 99, ICT93/15(Glutamine to Arginine at aa195), and ICT 358/15 showed a Glutamine to Lysine substitution at amino acid position 240 (Fig. 4).

### N-glycosylation

*N*-glycosylation is known to occur on Asparagine amino acid residues which occur in the Asn-Xaa-Ser/Thr stretch (where Xaa is any amino acid except Proline). While this consensus tripeptide (also called the *N*-glycosylation sequon) may be a requirement, it is not always sufficient for the Asparagine to be glycosylated. Two potential N glycosylation sites were identified in the sequenced F protein regions at positions 57–59, 172–174.

## Discussion

Human metapneumovirus (hMPV) is one of the leading causes of SARI in children and adults worldwide. Reliable diagnosis for clinical or research use is dependent on genome detection methods. Like other RNA viruses, hMPV exhibits a substantial rate of mutation due to its error-prone RNA polymerase leading to significant genetic diversity over time^15–19^. Throughout the study period, both hMPV genotypes A and B, including four known subtypes—A2a, A2b, B1, and B2 co-circulated round the year whereas subtype A1 was not detected in our samples^19–28^.

To date, studies on the epidemiological distribution and genetic diversity of hMPV have been reported mainly among hospitalized and outpatient children worldwide. Despite the significant burden of acute RTIs (Respiratory Tract Infections) due to hMPV, information regarding its seasonal distribution, circulating genotypes, and dynamics of disease transmission in tropical countries including Pakistan is currently limited^29^.

**Figure 5:**
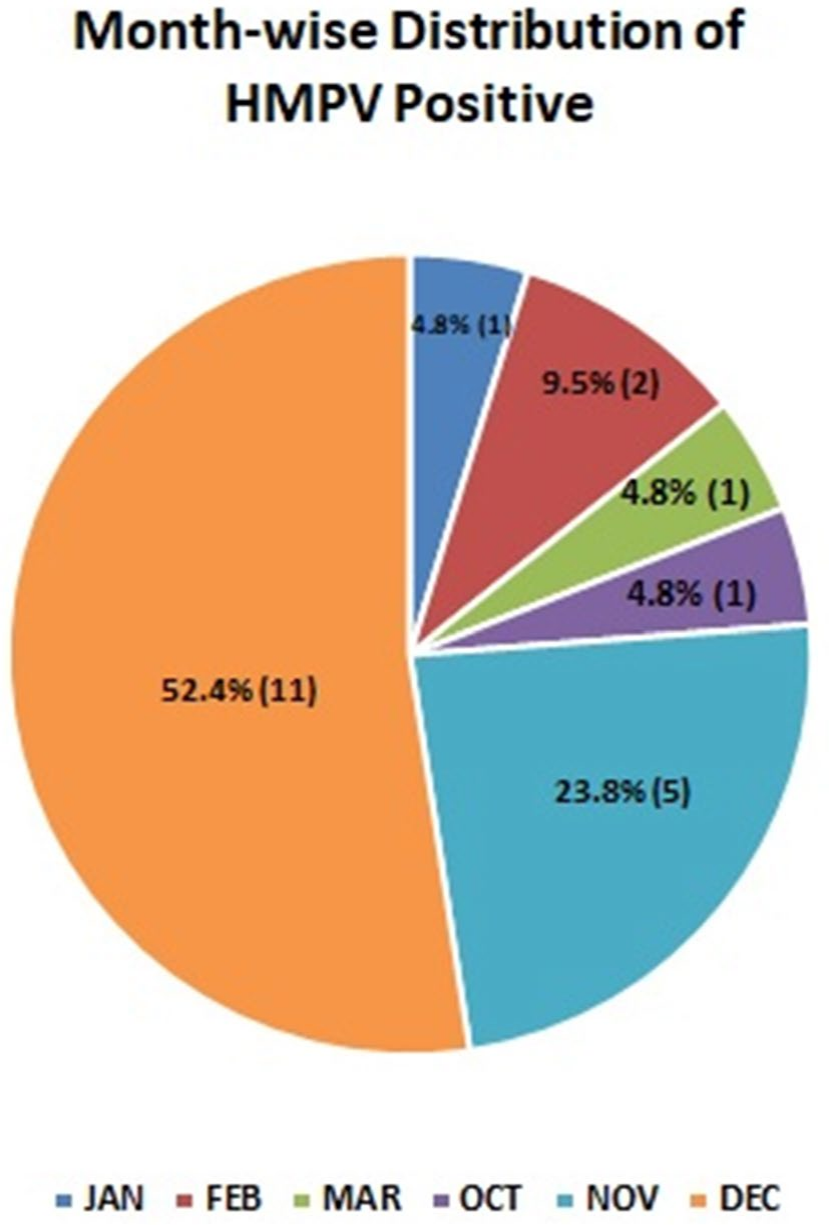
Month-wise distribution of hMPV positive samples.

In this study, we aimed to investigate the genetic diversity, seasonality, and transmission network of hMPV infections among children exhibiting SARI symptoms and reported at PIMS hospital in Islamabad, during January–December 2015. Genetic variation is a major factor that plays a key role in the positive selection of viral pathogens. The considerable genetic variability in the hMPV genome gives the virus a strong characteristic which renders it to circulate among the susceptible population and infect huge cohorts-mainly children. Based on F-gene sequence analysis, we found infection of both genetic groups of hMPV A (A2a, A2b) and B (B1 and B2) in the study subjects. These findings are consistent with data generated in other countries including India and South Africa^13,30^. Different sub-groups of hMPV detected in this study indicated variable frequency of infection during 2015 which implies that viral variants are temporally distributed (Fig. 5). Globally, the temporal trend of non-hMPV viral respiratory infections in children including RSV-A, RSV-B, Influenza-A, Influenza-B, adenovirus has been frequently noticed during the winter season with peaks in December and January^9^. A2a, A2b, B1, and B2 clusters have been found in this study which has previously been reported in different countries including South Africa, England, and Argentina. There is a study report from Germany which classified genotype A into A2a and A2b which is similar to our study^31^.

In study samples, genetic group B2 was predominantly found in 60% of the positive samples followed by sub-lineage A2b which has been found in 20% of hMPV positive samples. The sub-lineage A2a and A2b viruses which were found among 10% and 20% of the positive samples respectively clustered with those reported in Singapore, China, and India^22,30^. The genotype B1 was detected in 10% of the hMPV circulating strains and clustered closely with the strains reported in India^30^ and the USA (accession number KC562219). The B2 genotype which is the most dominantly present genotype found among samples positive for hMPV was clustered close to the strains reported in India^30^.

This study demonstrates that hMPV is one of the contributing pathogens which causes acute respiratory illness among children less than 5 years of age having a mean age of 2.6 ± 1.76 years. The positive rate of hMPV linked acute respiratory infections in our study was 16.5% which is quite higher than its prevalence in Iran (5.7%), Amman (6%), and Brazil (5.6%)^32^. In addition, our study reflects that most of the hMPV infections were reported in the first and last quarter of the year highlighting that the circulation of the virus is associated with the winter season (Fig. 5). Of note, this is the first report of molecular characterization of hMPV strains from Pakistan and documents the presence of A2a and A2b sub-lineages along-with genotypes B1 and B2 and warrants to scale-up surveillance initially at major children’s hospitals across the country to assess the disease burden and better understand its epidemiology and clinical significance.

### Limitations

There are certain limitations to this study. Samples were collected for 12 months and the seasonal pattern of respiratory viruses can vary from year to year. Multiple-year surveillance data is needed to reliably establish the seasonality of hMPV and other respiratory viruses. In resource-limited settings, conducting radiographic investigations are not convenient for all patients due to resource constraints.

## Supplementary Information

Supplementary Information 1.

Supplementary Information 2.

Supplementary Table 1.
