# Supplementary material for: Molecular detection and genetic characterization of human metapneumovirus strains circulating in Islamabad, Pakistan
Source: Sci Rep. 2022 Feb 18;12:2790. doi: 10.1038/s41598-022-06537-5 (PMC8857187; doi:10.1038/s41598-022-06537-5)
Supplement: Supplementary file 1 — Supplementary Information 1. [file 41598_2022_6537_MOESM1_ESM.docx]

**cDNA Synthesis Protocol**

| **REAGENTS** | **Concentration** | **Volume (20µl)** |
| --- | --- | --- |
| **dNTPs** | 25mM | 0.25 |
| **Random Hexamers** |  | 0.5 |
| **RNA** |  | 12.5 |
| **Heat** | 65^o^C | 5 minutes |
| **Ice Chill** |  | 5 minutes |
| **dH2O** |  | 1.0 |
| **RNase Inh** | 40U/ µl | 0.5 |
| **dTT** | 0.1 M | 0.7 |
| **RT Buffer** | 5X | 4.0 |
| **AMV RT** | 20U/ µl | 0.7 |
| **Temperature 1** | 25 ^o^C | 10 minutes |
| **Temperature 2** | 42 ^o^C | 50 minutes |
| **Temperature 3** | 70 **^o^C** | 5 minutes |

**PCR Reagents and Protocols**

| **PCR Reagents** |
| --- |
| 10X PCR Buffer (NH4)_2_SO4 |
| 25mM MgCl_2_ |
| 10mM dNTPs |
| 5U/µl Taq DNA Polymerase |
| cDNA template |
