# Supplementary material for: Molecular detection and genetic characterization of human metapneumovirus strains circulating in Islamabad, Pakistan
Source: Sci Rep. 2022 Feb 18;12:2790. doi: 10.1038/s41598-022-06537-5 (PMC8857187; doi:10.1038/s41598-022-06537-5)
Supplement: Supplementary file 2 — Supplementary Information 2. [file 41598_2022_6537_MOESM2_ESM.docx]

**PCR and Sanger Sequencing Reagents Details**

| **Reagents** | **Catalogue Numbers** |
| --- | --- |
| [**AgPath-ID™ One-Step RT-PCR Reagents**](https://www.thermofisher.com/order/catalog/en/US/adirect/lt?cmd=catProductDetail&showAddButton=true&productID=AM1005&_bcs_=H4sIAAAAAAAAAI1RUWuDMBD%2BNXmZWGKCzte2Uhgbq9TuuQQ9NRCNxLPFf78LkjL2MAbh7svl8t33%0AJXHCeF462yw1zhETWVSBu%2Bsa5j%2FqPeLE5J6JE63H47HDHtxgWz1T3tV2oPIyU4CRQm8HoGRdQ4c9%0ADoYYmJB%2B8RzdAn7PX72QFm%2BJ4Al1tzfBeSoS8ULlfVcq7OO3gnSgUw0weYjOI8QVwhRdrnF5vEQX%0AUB2M%2BD99cp%2FzoIlyrVAZ2wXFXxUF1WgHNRIyyOSpHhomC2os9DwZtVa4GrKV%2Be2GZVHpsTPwAXcw%0AR4XQWbdSB915h5WOE%2B8sO5cEW23QT8424Ek%2B1eA5fnhNg1uRygMTh%2BCY4ObZg%2BA6UF3XydMkz4Kf%0AFp41%2FfWwz29olZn9P3wDVM4dFw8CAAA%3D&returnURL=http%3A%2F%2Fwww.thermofisher.com%3A80%2Forder%2Fcatalog%2Fen%2FUS%2Fadirect%2Flt%3Fcmd%3DcatDisplayStyle%26catKey%3D101%26filterType%3D1%26OP%3Dfilter%26filter%3Dft_1201%252Ff_2005212*) | AM1005 (cat #) |
| **RT PCR Buffer 10x with MgCl** | ABI |
| **dNTPS** | 11-277-049-001-Roche |
| **Taq DNA Polymerase** | 10342-053 (10X buffer, MgCl), 100units-Invitrogen |
| **AMV Reverse Transcriptase** | 12328019, 15U/ul, 750units-Invitrogen |
| **RNAse Inhibitor** | 03-335-399-001-Roche |
| **QiaAmp Viral RNA Minikit** | 52904-Qiagen |
| **BigDye Terminator v3.1 Cycle Sequencing Kit** | 4336935 Applied BioSystems |
