# Supplementary material for: Molecular detection and genetic characterization of human metapneumovirus strains circulating in Islamabad, Pakistan
Source: Sci Rep. 2022 Feb 18;12:2790. doi: 10.1038/s41598-022-06537-5 (PMC8857187; doi:10.1038/s41598-022-06537-5)
Supplement: Supplementary file 3 — Supplementary Table 1. [file 41598_2022_6537_MOESM3_ESM.docx]

**Molecular detection and genetic characterization of human metapneumovirus (hMPV) strains circulating in Islamabad, Pakistan**

Supplementary Table 1. Primers and Probes

| **Primer Name** | **Sequence (5’-3’)** | **Target Gene** |
| --- | --- | --- |
| **HMPVFextF^16^** | 5’-ATGTCTTGGAAAGTGGTG-3’ | F |
| **HMPVFextR^16^** | 5’-CCATGTAAATTACGGAGCT-3’ | F |
| **HMPVFintF^16^** | 5’-TCATGTAGCACTATAACT-3’ | F |
| **HMPVFintR^16^** | 5’-TCTTCTTACCATTGCAC-3’ | F |
| **HMPV(FOR)^43^** | 5’-CAAGTGTGACATTGCTGAYCTRAA-3’ | F |
| **HMPV(REV)^43^** | 5’-ACTGCCGCACAACATTTAGRAA-3’ | F |
| **HMPV(P)^43^** | 5’-FAM-TGGCYGTYAGCTTCAGTCATTTCAACAGA-3’ | F |
